# Image Based Mango Fruit Detection, Localisation and Yield Estimation Using Multiple View Geometry

**DOI:** 10.3390/s16111915

**Published:** 2016-11-15

**Authors:** Madeleine Stein, Suchet Bargoti, James Underwood

**Affiliations:** 1 Division of Automatic Control Department of Electrical Engineering, Linköping University, Linköping SE-581 83, Sweden; madst314@student.liu.se; 2 The Australian Centre for Field Robotics (ACFR), Department of Aerospace, Mechanical and Mechatronic Engineering (AMME),The University of Sydney, Sydney, NSW 2006, Australia; suchet.bargoti@sydney.edu.au

**Keywords:** computer vision, agrivision, fruit detection, yield estimation, field robotics

## Abstract

This paper presents a novel multi-sensor framework to efficiently identify, track, localise and map every piece of fruit in a commercial mango orchard. A multiple viewpoint approach is used to solve the problem of occlusion, thus avoiding the need for labour-intensive field calibration to estimate actual yield. Fruit are detected in images using a state-of-the-art faster R-CNN detector, and pair-wise correspondences are established between images using trajectory data provided by a navigation system. A novel LiDAR component automatically generates image masks for each canopy, allowing each fruit to be associated with the corresponding tree. The tracked fruit are triangulated to locate them in 3D, enabling a number of spatial statistics per tree, row or orchard block. A total of 522 trees and 71,609 mangoes were scanned on a Calypso mango orchard near Bundaberg, Queensland, Australia, with 16 trees counted by hand for validation, both on the tree and after harvest. The results show that single, dual and multi-view methods can all provide precise yield estimates, but only the proposed multi-view approach can do so without calibration, with an error rate of only 1.36% for individual trees.

## 1. Introduction

Current industry practice to estimate the number of fruit in a commercial orchard block involves manually counting the average number of fruit on several trees and multiplying by the total tree count [1]. The amount of fruit on each tree can be highly variable, and so, the total estimates are often inaccurate. Additionally, fruit counts should ideally be assessed at several times during crop growth, which today is too labour intensive, costly and inefficient for farmers [1]. After harvest, larger commercial operators can use machinery in the packing-shed to weigh and count the fruit, which provides size or weight distributions from individual orchard blocks retrospectively, as long as the book-keeping to map fruit-bins to blocks was done correctly during the harvest. Both methods give data about entire orchard blocks and cannot be used to map the distribution of yield spatially within a block, which is necessary for precision agriculture management per tree. By contrast, machine vision systems facilitate counting fruit on each tree, in a manner that is efficient at the whole-orchard scale. This in turn will enable more frequent and more accurate fruit mapping, to allow commercial orchards to adopt precision agriculture techniques to address the needs of each tree and to plan ahead for harvest labour, packing-shed operations and marketing.

There is substantial prior work in robotics, sensing and machine vision for fruit detection in orchards, for applications including yield mapping and automated harvesting [2,3]. Methods include a variety of platforms, such as manned and unmanned ground vehicles (UGVs) [4,5,6,7,8], unmanned aerial vehicles (UAVs) and hand-held sensors [9]. Different types of imaging sensors have also been used, including “standard” (visible light) cameras and stereo, near infra-red, long wave thermal infrared cameras and LiDAR [2,3,4,9,10,11,12,13,14]. Standard cameras are a common choice, due to their low cost, the richness and comparatively high resolution of the data they provide, as well as the familiarity to the machine vision community in terms of data acquisition and processing techniques [12].

Much of the research has focused on improving the accuracy of fruit detection within imagery; however, the relationship of image-based fruit counts and the actual number of fruit on the tree is challenged by visual occlusion, which cannot be addressed by improved classification performance. This is a problem that is mentioned in the vast majority of the literature. Progress over the last five years from the machine vision community with convolutional neural networks (CNNs) has led to highly accurate fruit detection in colour imagery [15,16,17], and so arguably, the focus should shift towards fruit counting systems, which are designed to acquire and process orchard imagery in such a way that delivers the highest accuracy compared to the actual field and harvest fruit counts.

Systems that acquire a single image per tree (or one image from both inter-row perspectives) require either that all fruit are visible from only one or two views or that there is a consistent, repeatable and modellable relationship between the number of visible fruit compared to the total on the tree. In the latter case, a process of calibration to manual field-counts can be done, which has proven to be accurate for some canopy types, including trellised apple orchards [5,6,15], almond orchards [7] and vineyards [18,19]. The calibration process requires manual field or harvest counts, which is labour intensive and would ideally be repeated every year. Calibration was validated from one year to the next by Nuske et al. [18], but is not guaranteed to hold over multiple years of canopy growth, nor for different fruit varieties and tree canopy geometries. Furthermore, the process is subject to human error, which propagates to all subsequent yield estimates.

Another approach is to acquire and process images from multiple viewpoints to combat the effect of occlusion from any one perspective. For relatively open canopies where the fruit is not heavily bunched (e.g. apples, mangoes, citrus), this has the potential to enable every piece of fruit to be directly observed and counted. For heavily bunched fruit, such as grapes, this may only improve performance if the camera can be flexibly positioned in three dimensions around the bunches. To count fruit seen from multiple views, each fruit must be uniquely identified, associated and tracked between images to avoid over-counting. There are several examples of multi-viewpoint fruit tracking in the literature for different types of fruit: peppers have been tracked and counted by using a statistical approach to cluster repeated observations [20]; optical flow has been used to associate fruit in subsequent images for citrus [9]; pineapples were tracked and counted over image sequences using feature matching and structure from motion (SfM) [21]; stereo vision has been used to detect and associate apples [4,22]. Amongst these examples, quantified comparisons to the true number of fruit counted in the field are only given in two cases [4,21], and both show a near unity relationship, suggesting that multi-view fruit tracking has promise. In apples, the relationship held when fruit was thinned, but under-counting occurred when apples were left in bunches [4].

In this paper, we investigate the use of multiple view-point fruit detection, tracking and counting as a means to measure and map the quantity of fruit in a mango orchard. Mangoes are grown on individual trees with three-dimensional (3D) canopies, in contrast to the 2D apple trellis fruit-wall that was studied by Wang et al. [4]. The 3D canopies present distinct challenges for fruit detection and tracking: the large detection volume is difficult to illuminate uniformly with strobes or by choosing the time of day of the scan; the distance between the sensor and fruit is variable, causing appearance variation due to scale and optical flow variation as a function of range; the complex canopy geometry can cause fine-grained patterns of occlusion between subsequent monocular frames or stereo pairs, and that combined with the sensor-to-target range variation can cause unpredictable failures in dense stereo depth estimation. To meet these challenges, we use a state-of-the-art CNN approach for fruit detection in individual monocular images, which can handle the variability in scale and illumination of the fruit. We couple this with a monocular multi-view tracking method that uses a global positioning inertial navigation system (GPS/INS) to directly provide the absolute and relative camera poses for each image, instead of relying upon image feature extraction and matching between frames as mentioned, but not detailed by Das et al. [9]. As a result, and distinct from Song et al. [20], Roy et al. [22], we are able to perform frame-to-frame fruit tracking using epipolar geometry combined with the “Hungarian” algorithm [23] for optimal tracking assignment. An advantage of this framework is that the fruits are detected, matched and tracked purely in the image-domain, meaning that we are not restricted by stereo or SfM depth estimation accuracy for tracking or counting. Once tracked, we triangulate the fruit in order to calculate spatial statistics. The triangulation accuracy is subject to matching and localisation errors; however, this occurs after the image-based data-association step and does not affect tracking (and therefore, counting) accuracy.

The fruit tracking, counting and mapping system is combined in a novel way with a LiDAR segmentation approach that was previously developed for almonds [7,24], which segments datasets from entire orchard blocks into separate rows and individual trees. The novel extension uses the GPS/INS navigation data to project thinned segmented LiDAR points into every image, to audit the images regarding which specific trees are visible. The full LiDAR tree geometry is then projected into the image space in correct depth perspective order to automatically create a tree identification image mask. Detected fruit can then be associated with their corresponding specific row and tree identifiers by a process of masking and voting. The association step allows the calculation of fruit statistics per tree, as well as per row or for the whole orchard block. The same system also allows single and dual opposing view-based estimates to be associated with individual trees, which has allowed a comprehensive experimental comparison of the performance of single, dual and multi-view fruit counting, which to the authors’ knowledge has never been done before. The experiments, results and discussion will be of value to other researchers and developers in assessing the relative merits of the different methods.

## 2. Materials and Methods

**Figure 1:**
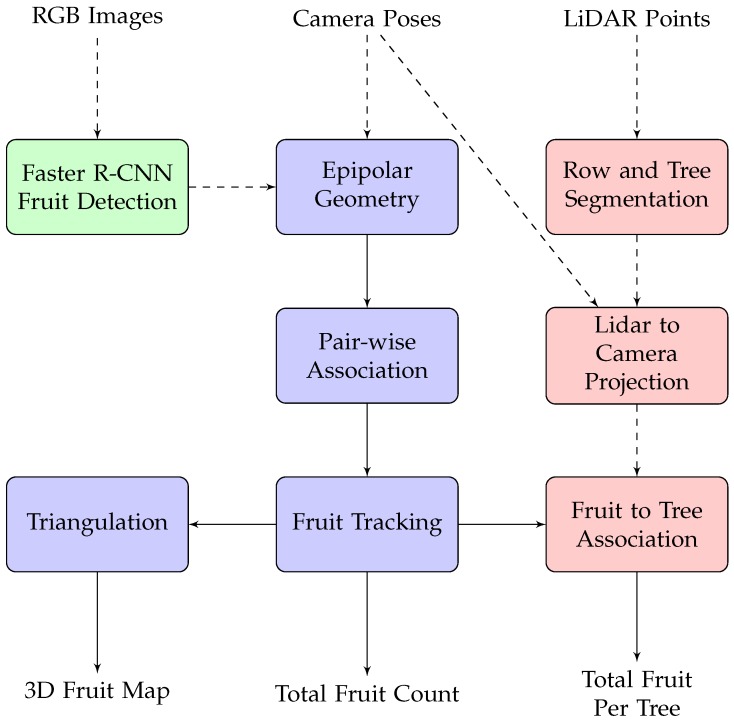
Flow chart of the proposed algorithmic pipeline. Fruit is detected within each RGB image using faster R-CNN. This is the only step that uses the image data (shown in green). Using the camera poses provided by the navigation system, the the fruit is associated and tracked through sequences of multiple images from multiple viewpoints and triangulated to locate in 3D, using a sequence of geometric operations (blue). LiDAR data (red) are used to associate the fruit with the corresponding tree.

This section describes the unmanned ground vehicle (UGV) system that was used to acquire sensor data, the mango orchard where experiments were performed and the algorithmic pipeline that is proposed to process the data to detect, track and count the fruit and to associate the counts to specific trees. An overview of the pipeline is illustrated in Figure 1. Fruits are detected in individual colour images using faster R-CNN (FR-CNN) [25], a state-of-the-art object detection network. The camera poses provided by a navigation system are used to calculate the epipolar geometry between subsequent image pairs, which allows robust pair-wise association of the fruit. Tracking is performed by linking the chain of pair-wise associations, which then enables the unique count and triangulation of the fruit. Separately, LiDAR data are segmented to identify individual trees, which are geometrically projected to the image-domain to form tree-identification masks to associate each fruit with its corresponding tree. This section describes the equipment and all of the steps in Figure 1 in detail.

### 2.1. Data Collection

**Figure 2:**
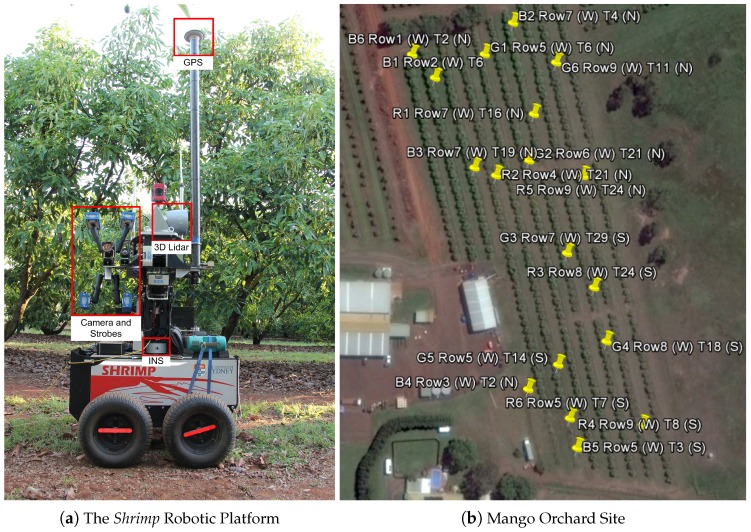
The robotic platform Shrimp (**a**) with labels to indicate the locations of the sensors used in this paper; a map of the mango orchard test block (**b**), with yellow pins marking the 18 trees that were selected for ground-truth field and harvest fruit counts.

Data were collected using an unmanned ground vehicle (UGV) called “Shrimp”, developed at the Australian Centre for Field Robotics (ACFR), which is equipped with a colour camera and strobes, a 3D LiDAR and a global positioning inertial navigation system (GPS/INS) as indicated in Figure 2a. The navigation system is capable of real-time-kinematic (RTK) correction, but this was not applied due to communications constraints. Colour images were acquired at 5 Hz using a Prosilica GT3300C camera [26] with a Kowa LM8CX lens [27], synchronised to four Excelitas MVS-5002 strobe lights [28]. The camera was directly connected to a dedicated Ethernet port, and images were timestamped with the system clock on arrival. The images were 3296 × 2472 pixels each (8.14 megapixels). This configuration provided approximately 25 viewpoints of each side of the tree, when passing at 1.4 m/s. LiDAR data were captured from a Velodyne HDL64E 3D LiDAR [29], configured to spin at 10 Hz with a data rate of 1.3 million points per second. Contrary to typical practice, the Velodyne was mounted ‘sideways’ to capture the full height of the tree canopies. The Novatel SPAN system combines an OEM3 GPS receiver with an IMU-CPT inertial measurement unit [30], to provide six degree-of-freedom location and orientation of the platform at 50 Hz.

The data were collected from a single mango orchard block at Simpson Farms in Bundaberg, Queensland, Australia. This was done on 24 of November 2015, which was deemed by farm management to be the earliest time before harvest where no further significant fruit drops would occur. Additionally, 18 trees were selected for manual ground-truth counting, which was done in the field with fruit still on the tree and also in the packing shed after the fruit were harvested. The trees were selected to include six low, medium and high vigour trees according to their normalised difference vegetation index (NDVI) values measured from multi-band satellite data. The field counts were performed in mid December 2015, while the harvest counts were performed at harvest on 28 of January 2016. Harvest counts are likely to be more accurate than field counts, because the fruit are picked until none remain, and counting can be done more methodically with the fruit removed from the tree. Additionally, considering the timing, all comparisons in this work to the ground-truth harvest data are indicative of predictive yield estimation, two months prior to harvest.

The block comprised 10 rows, with a rectangular cut-out where a shed is located, as seen in Figure 2b. The total area of the block was 2 ha, and the average row length was 240 m. The 18 trees are marked with yellow pins and annotated by their orchard row (*r*) and tree number (*t*), counted from north or south (n/s). All of the 522 trees were scanned from both sides, by traversing every row in both directions while continuously recording the sensor data. The total trajectory length was approximately 5 km. At 1.4 m/s, this would be covered in one hour, but it took approximately 3 h, including scanning repeated rows and periodically checking the integrity of the data in the field. Shrimp is powered by a bank of lithium polymer batteries, which allow continuous operation for approximately two hours. They were hot-swapped once (without powering down the robot) in the field. Shrimp was built in 2009, whereas our more recent robots, including Ladybird and Rippa, feature solar charging and larger battery capacity, meaning they can run continuously without charging for multiple days [31].

The field and harvest counts are presented in Table 1. Two of the trees were excluded from further analysis: r5t14 had a discrepancy between ground-truth field and harvest counts, and r3t6n was excluded because it was completely occluded by a neighbouring non-mango tree. After this harvest season, the farmer removed all trees south of the shed in the first three rows (including r3t2n and its occluding neighbour), as this area contained some non-mango trees and was uneconomical to manage. As can be seen in Table 1, the remaining counts are similar, so the harvest counts were used to validate the performance for harvest yield estimation.

### 2.2. Fruit Detection

The fruit detection system utilises the FR-CNN framework, which is a state-of-the-art region-based CNN detector [25]. The network takes in as input an image of arbitrary size (with constraints of computational hardware) and returns a set of object bounding boxes and associated object probabilities. Recent work has demonstrated its use for single image fruit detection over a variety of fruits ranging from sweet pepper and rock-melon in greenhouses, strawberries, apples, avocados, mangoes and oranges from Google Image searches [17] and apples, mangoes and almonds from outdoor orchard scenes [16].

The network is composed of two modules: (1) a region proposal network (RPN), used for the detection of regions of interest (RoIs) in the image (i.e., fruits), followed by (2) a classification module, which classifies individual regions and regresses a bounding box around the fruits. The input images are initially propagated through a deep CNN (VGG16 [32] used in this paper), containing convolutional and fully-connected layers. The output from the final convolutional layer is a high dimensional feature map, which is then connected to two sibling layers. These layers define the RPN, and they detect and classify RoIs in the image. The RoIs are subsequently propagated through the fully-connected layers, denoted as the FR-CNN layers, which refine their object probability and associated bounding box label. The output from the network is a set of bounding boxes with fruit probabilities. To these, a probability threshold is applied, followed by non-maximum suppression (NMS) to handle overlapping detections.

Ground truth fruit annotations on the image data are required to train the CNN detector. For this, a learning dataset was constructed by randomly sampling 1500 crops of 500×500 from the collection of ∼15,000, 8.14 megapixel images over the orchard block (equating to 0.3% of the entire image data), as done by Bargoti and Underwood [16]. Randomly sampling subsets of the dense image data allowed the learning dataset to capture the scene variability over the orchard block. The small size of the cropped images also enabled learning deep CNN on a GPU, given their memory constraints. Individual fruits were annotated using rectangles, specifying their location and size. The labelling was exhaustive, annotating all fruits that could be identified by the human eye in the images. Fruits appearing on trees in background rows were ignored. Contrast and brightness adjustments to the images while labelling made it possible to identify fruit that was difficult to differentiate from the background in shadowed regions. Our python based annotation toolbox is publicly available [33]. In order to evaluate the impact of fruit detection accuracy towards yield estimation, images captured from each side of the 18 target trees from the previous section were also manually annotated with fruit labels. This is denoted as the dual-view hand labelled dataset and was not used for training the CNN fruit detector.

All network and learning hyper-parameters were fixed to the configurations used by Bargoti and Underwood [16]. In order to test the performance of the single-image mango detection system, the set of 1500 labelled images was split into 1000-250-250, representing the training, validation and testing sets. Detection performance was evaluated using the fruit F1-score, where the class threshold was evaluated over the held out validation set. A fruit detection was considered to be a true positive if the predicted and the ground-truth bounding box had an intersection over union (IoU) greater than 0.2. This equates to a 58% overlap along each axis of the object, which was considered sufficient for a fruit mapping application. For example, with higher thresholds (such as 0.5 used in PASCAL-VOCchallenges [25]), small errors in detections of smaller fruit cause them to be registered as false positives. A one-to-one match was enforced during evaluation, penalising single detections over fruit clusters and multiple detections over a single fruit. As a result, the mango detection performance over the test set was F1=0.881. Single-image fruit detection is an important component of this work, but is not the experimental focus. The reader is referred to Bargoti and Underwood [16] for a detailed analysis of fruit detection performance.

To perform fruit detection on the raw images spanning the entire block, a new network was trained using all 1500 images in the learning dataset. During inference, to accommodate for the large test image size (3296 × 2472), we utilised a tiled FR-CNN predictor [16]. The approach first performs detection using sliding windows, followed by applying a class probability threshold and NMS over the entire raw image.

### 2.3. Tree Segmentation

**Figure 3:**
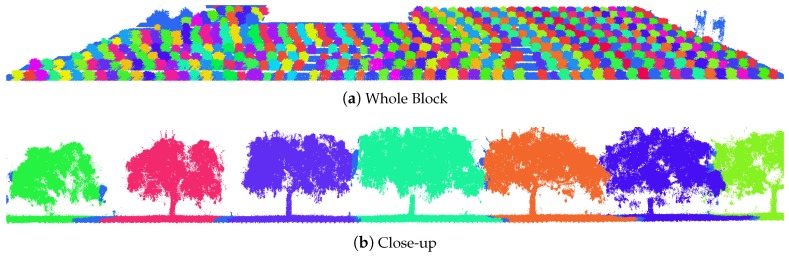
Segmented mango-tree LiDAR data, coloured by the tree count from the north headland to the right of the image. The whole block is shown in (**a**); and a close-up view of several trees in (**b**). Light blue bands indicate the boundary between trees.

During data collection, the vehicle was driven straight down the centre of each row, with a right-angle on-the-spot turn at the headlands. This created a salient marker in the GPS/INS data, to automatically detect the entry and exit times for each row. These times were used to select LiDAR and imagery corresponding to each side of each row. The trees were segmented in the LiDAR data using a hidden semi-Markov model (HSMM) that was previously developed for citrus [34], almond [24] and trellised apple [35] trees. Any incorrect tree segmentation boundaries were manually corrected, after which the trees were automatically labelled by their count from the north and south headland to match the field protocol. There were typically one or two segmentation errors per row, which took approximately 2 minutes each to manually remove. The primary cause of the errors was that we placed a ladder with a calibration target between trees (not used in this work), which caused ambiguity when segmenting the adjacent tree. The segmented 3D LiDAR data are illustrated in Figure 3. This approach also allows the calculation of canopy volumes from the LiDAR geometry, by removing ground points, voxelising the canopy points and summing the voxel volumes as described for almond trees in our prior work [7].

The complete three-dimensional (3D) tree database was then used to create an inventory of which trees were seen in which images from the free-running video camera, as follows. All images for one side of one row were selected according to the entry and exit times. Segmented LiDAR data for that row were voxelised with a resolution of 20 cm and then thinned to 5%, to reduce computation time. The thinned data were geometrically projected into the camera frame [36]. For each image, if a single thinned LiDAR point for a particular tree was present within that frame, then the image was deemed to “contain” that tree. Given the geometries of the camera, platform and orchard, there were either none, one, two or three foreground trees in a single image. Trees in adjacent rows were often visible behind the foreground trees, but these were correctly suppressed in the image contents because the LiDAR data and imagery were processed strictly one row at a time.

**Figure 4:**
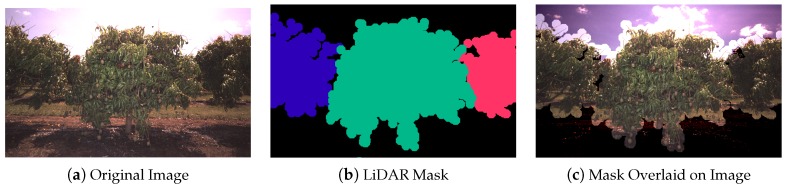
Image masks generated from projected LiDAR data: (**a**) the original image; (**b**) the projected LiDAR mask coloured by tree count; and (**c**) overlaid with transparency to highlight the accuracy of the alignment. The boundary is wider than the tree due to dilation.

Image masks were then created from the LiDAR data to specify which pixels within each image belong to which tree. The process was adapted from the masking method performed for almond trees [7] to cater to multiple trees per image as required by the multi-view application. For each image, all of the non-voxelised, non-thinned, LiDAR points corresponding to all trees contained in that image were projected to the image [36]. To preserve the effect of occlusion from the perspective of the camera (which differs from the perspective of the LiDAR), the points nearest to the camera (within a 20 pixel lateral radius) were prioritised in the mask. These points were then dilated one tree at a time by the same pixel radius and layered into the mask in order of furthest to nearest tree. An example of a LiDAR-generated image mask is shown in Figure 4.

For each detected fruit in an image, the mask was used to assign that fruit to the corresponding tree. When a piece of fruit near the boundary of two trees is tracked over multiple images, it may be assigned inconsistently. In this case, assignment was decided by the maximally-occurring correspondence vote.

To compare the multi-view approach to single and dual-view methods, where only one image from one or both sides of the tree are used, the best single image of each side of each tree was automatically determined as the one where the mask centroid was closest to the centre of the image.

In this system, LiDAR-based image masking is only necessary to associate fruit counts to individual trees. This was necessary in this study to compare to the ground-truth counts from 16 specific trees, but is also likely to be useful to facilitate precision management decisions at the scale of individual trees. The masking is, however, not strictly required for fruit detection, tracking, localisation, yield mapping or for retrieving fruit tallies and statistics per row or per block, since these operations do not require strict per-tree association. If per-tree association is not required, the dependency on LiDAR data is removed from the system.

### 2.4. Fruit Tracking, Localisation and Counting

**Figure 5:**
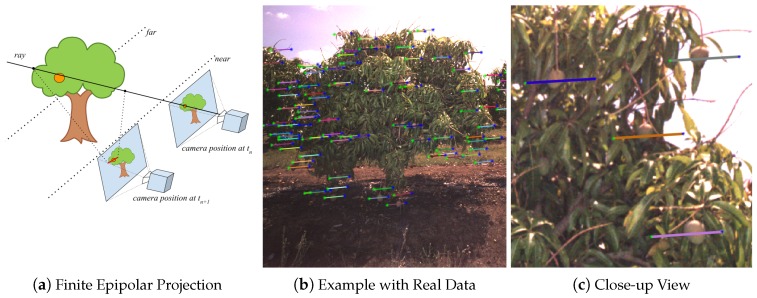
An illustration (**a**) of how epipolar line segments were calculated. A ray is projected from the camera centre corresponding to the first image at $t_{n}$, through the location of a mango in the image-plane. The ray is clipped at two distances (constant for the whole orchard). The start and end points of the ray are then projected into the second image-plane at $t_{n+1}$. An example with real data is shown in (**b**) with a close-up in (**c**).

Fruits detected in each image (as described in Section 2.2) must be associated and tracked throughout the sequence of all images in which they are seen, to avoid counting the same fruit multiple times. This section describes how fruits were associated from one image to the subsequent image in order to count them uniquely. For each fruit detected in the first image, the epipolar line corresponding to the centroid was projected into the subsequent image. For each detected fruit, a ray was projected from the camera focal point of the first image at $t_{n}$ through the location of a detected mango in the image-plane. The ray was then clipped at a near and far distance of 2.5 m and 8 m, which was a conservative camera-to-fruit distance constraint for the whole orchard. It might be possible to use the LiDAR data to constrain the range specifically for each fruit, but we opted to minimise the dependency on LiDAR for tracking. We found that the constant conservative bounds were sufficient. The start and end points of the ray were then projected into the second image-plane at $t_{n+1}$. If infinite epipolar lines were used, they would span the entire image, intersecting multiple mangoes, making the correct correspondence ambiguous. The conservative range constraint resulted in finite lines and less ambiguity. The projection requires the poses of the camera when the two images were captured, which was supplied by the GPS/INS trajectory data. The process is illustrated in Figure 5. Each line in Figure 5b shows the region in the image where a fruit that was detected in the previous image must lie in this image, if it is still visible.

Fruit are independently detected in the second image also. Thus, a data association step is required to match $t_{n+1}$ detections to the finite $t_{n}$ epipolar projections. To achieve this, an association cost was calculated between all permutations of detection centroids and epipolar line segments. The perpendicular distance forms the basis of the cost, with two additional constraints: if the perpendicular distance was greater than a threshold or if the centroid lay beyond the ends of the finite epipolar line, then the association cost was set to infinite. In the implementation, a finite number that is larger than the diagonal size of the image was used. This is the only tunable threshold in the multi-view tracking system. Thresholds across the entire permissible range were evaluated to select the most appropriate value, and the algorithm was not found to be sensitive to this parameter, as described in detail in Section 3.

New fruit appear, and previously-seen fruit become occluded, so a one-to-one match is not expected. For *i* points and *j* lines, this forms a rectangular matrix of association costs, and the Kuhn–Munkres or “Hungarian” algorithm was used to find assignments between detections and finite epipolar lines that minimised the total association cost [23,37,38]. In addition to tracking the fruit, the approach also rejects single occurrences of false positive detections, where they are not matched to another detection in a previous or subsequent image. The approach to associate the detections from $t_{n}$ to $t_{n+1}$ was then iteratively applied throughout the entire image sequences for each row, to assign a unique integer identifier (id) to each piece of fruit. The fruit was tallied by counting the number of unique ids per tree, per row and in total for the whole orchard block.

The locations of each mango were determined by triangulating all tracked observations. Each observation forms an infinite ray in three-dimensional Cartesian geo-coordinates. In Section 2.4, the rays are constrained and finite, whereas for triangulation, they are effectively unconstrained. In theory, rays from a single fruit would intersect at a single location, but that is rarely true in practice due to several sources of error, including intrinsic and extrinsic sensor calibrations, time synchronisation errors between sensors [39] and the inexact detection centroids of the fruit. Instead, for each fruit, the shortest line segment was calculated between all pair-wise permutations of observation rays, and the mid-points of those segments were averaged to estimate the 3D fruit location. The height of the fruit above the local ground was calculated as the vertical distance between the triangulated geolocation of the fruit and the nearest point on the LiDAR surface elevation map. Finally, the time synchronisation error between the camera and GPS/INS was estimated by optimising it against the fruit association cost, on the assumption that the average distance between detected fruit and epipolar projections would be lowest when the correct, but unknown time offset was applied.

The system provides a complete list of every piece of fruit, including its 3D geolocation and local height above the ground and its association with a specific row and tree number. From this, many different spatial statistics can be derived, including yield maps, histograms or tallies of the counts or spatial properties per tree, row and orchard block.

## 3. Results

This section quantifies the performance of the system, by comparing the counts for 16 specific trees against ground-truth field and harvest fruit counts and by cross comparison for all 522 trees in the orchard block. The stability of the system was evaluated against different values for the only tunable parameter (the pixel distance threshold), and both the distance threshold and the time-offset between the camera and navigation system were optimised. The fruit counting precision, accuracy and repeatability of the multi-view system were quantified and compared to the single and dual-view approach. Spatial properties, including the triangulation, height above ground and fruit clustering, were qualitatively assessed.

### 3.1. Threshold Selection

**Figure 6:**
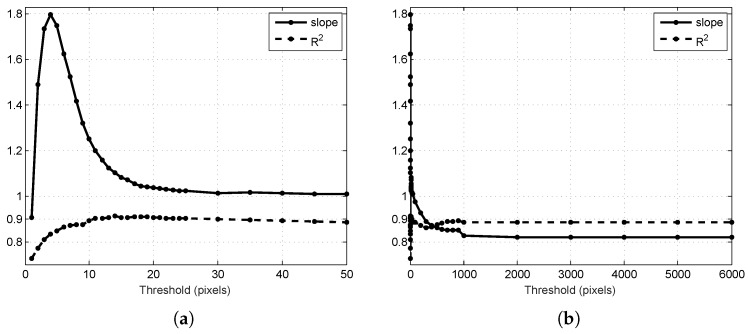
Performance of the algorithm for different threshold values: (**a**) in the region of reasonable values; (**b**) for ranges up to the size of the images. In both cases, values nearer to one indicate better performance. Higher $R^{2}$ values indicate better precision, and a slope nearer to one indicates better accuracy, compared to the harvest fruit counts for the 16 target trees.

The system described in Section 2 was used to estimate the number of mangoes on 16 ground-truth trees, over the range of permissible thresholds from one to 6000 pixels (which is larger than the diagonal image size). For each threshold, the least squared error linear model with clamped zero offset (y=mx) was fitted between the multi-view estimate and harvest counts. The performance was summarised by the slope *m* and coefficient of determination ($R^{2}$) of the model. The $R^{2}$ value varies from zero to one, where one indicates a perfect linear relationship to the ground-truth or perfect precision. The slope is unrestricted and represents the percentage of fruits the are counted. The closer the slope is to one, the higher the accuracy. The results are shown in Figure 6.

For very low threshold values (<15), fruit tracking is effected in two competing ways: (1) fruit is only counted if it is associated in at least one pair of images; therefore, low thresholds can increase the number of fruits that is missed completely, driving the count down; (2) when a track is established, low thresholds increase the probability that the track will terminate in subsequent images and then re-initialise, which drives the count up. For low thresholds, these competing forces caused instability in the slope and a lower $R^{2}$ value, as seen in Figure 6. For threshold values above 15, both the slope and $R^{2}$ reached a stable plateau near the optimal value of one for both measures. The graph shows that the algorithm is not sensitive to the tuned parameter value, as long as it is above the noise-floor of the system. The noise floor depends on a number of factors, including the accuracy of the navigation data, the velocity and rotation rates of the platform, the accuracy of time synchronisation between the navigation and camera data, the distance to the fruit and the resulting size and separation of the fruit in pixels. A reasonable estimate for this system is between four and 12 pixels from Section 3.2.

For large thresholds (>100 pixels), the precision remained high ($R^{2}∼0.9$), but the slope decreased to value of 0.82. With very large thresholds, the algorithm is able to match a fruit to any other in the entire image. The optimal association still robustly matches the lowest distance associations (and therefore, often the correct associations), allowing a reasonable correlation to the true counts. Under-counting occurred because whenever a mango disappeared from view, it was matched to a distant neighbour instead of forming a new track. From the graph, a threshold of 30 pixels was selected, corresponding to $R^{2}=0.90$, m=1.014.

### 3.2. Camera and Navigation Timestamp Offset Correction

**Figure 7:**
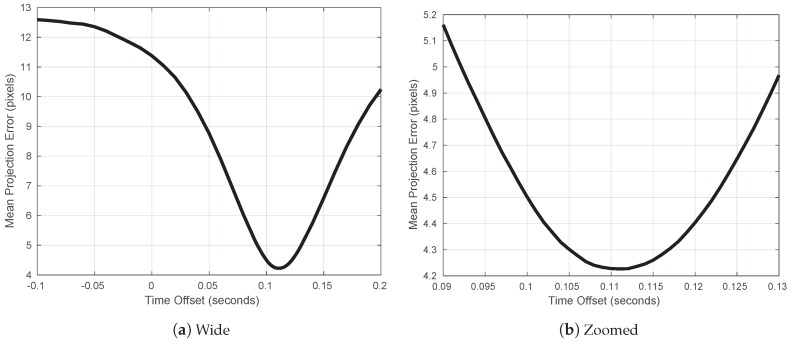
The mean pixel association error for all fruits in the 16 target trees, as a function of an additive time offset between the navigation data from the GPS/INS and the images. A clear global minimum is visible. The peak occurs for a time offset of 0.111 s, with a minimal average error of 4.2 pixels.

**Figure 8:**
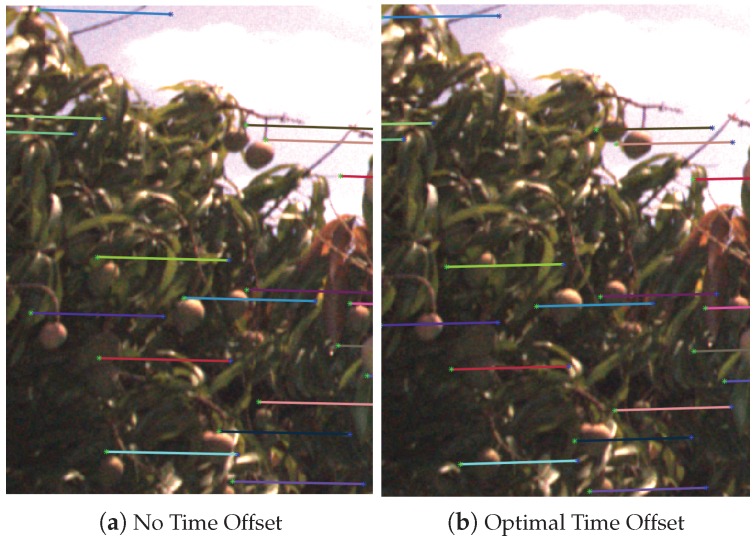
An example of projecting epipolar line segments from one image to the next, with and without the optimised time offset: (**a**) no offset is applied; (**b**) the optimal offset of 0.111 s is applied. The average error reduced from 11.4 to 4.2 pixels, which agrees with the magnitude of errors in this example.

The image timestamps were recorded as the time-of-arrival of the image into computer memory, according to the computer system clock. To compensate for the time offset error between the asynchronous image timestamps and the GPS/INS data (which was precisely synchronised to the GPS clock), a variable additive offset was applied to the image timestamps. Time offsets were evaluated between −0.1 and +0.2 s. For each offset in this range, the fruit were tracked separately on both sides of the 16 target trees and the average fruit association pixel distance was calculated. This could be performed on any sequence or set of sequences of images and does not have to be done for individual trees. We performed it on a number of specific trees because this was easy to do with our framework, and using multiple sequences allowed us to confirm that the time offset was consistent at different times throughout the dataset. The result is shown in Figure 7, indicating a clear global minimum at 0.111 s, with an average association error of 4.2 pixels. To test the stability of the time offset at different times throughout the whole dataset, the same process was performed for each side of each of the 16 trees separately (resulting in 32 graphs similar to Figure 7). The mean minima occurred at 0.111 s with a standard deviation of 0.0044 s, which indicates that the time offset was stable at the 32 different times throughout the data logging period. The image size of 98 million bits divided by the gigabit Ethernet speed ($10^{9}$ bits/s) gives an expected image delay of 0.098 s, which is close to the observed time offset. The time optimisation reduced the mean error from 11.4 pixels down to 4.2 pixels. The benefit of the optimisation was also inspected visually for several trees, and an example is shown in Figure 8. Figure 8a shows the projections with a time offset of zero, whereas Figure 8b uses the optimised value. It is clear to see how the optimised time offset improves the projection accuracy.

### 3.3. Fruit Counts

**Figure 9:**
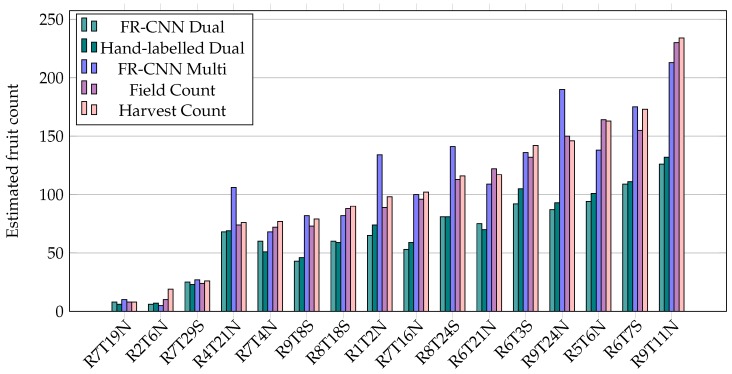
Estimated dual and multi-view counts for 16 target trees, with manual field and harvest ground-truth data. FR-CNN and hand-labelled dual show the dual-view method using the machine and hand labelled detections, respectively. FR-CNN multi refers to the proposed multi-view approach. Field counts were gathered by manually counting the fruit on the tree, in the field, just before harvest. Harvest counts were obtained by manually counting the fruit after removal from the tree.

Fruit counts were estimated for every individual tree in the orchard block using the multi-view algorithm from Section 2. The counts corresponding to the 16 target trees were extracted automatically by row and tree number for comparison to the ground-truth field and harvest counts. The correct correspondence was manually verified by confirming the presence in the images of a blue tag that was tied around the target-tree trunks in the field. Additionally, the mangoes detected in the two single images from the opposite sides of each target tree, within the LiDAR mask, were summed. This was done using both the hand-labelled images and FR-CNN detections, to compare the single, dual and multi-view approaches. The estimated fruit counts from the dual and multi-view methods are shown in Figure 9, together with field and harvest counts.

**Figure 10:**
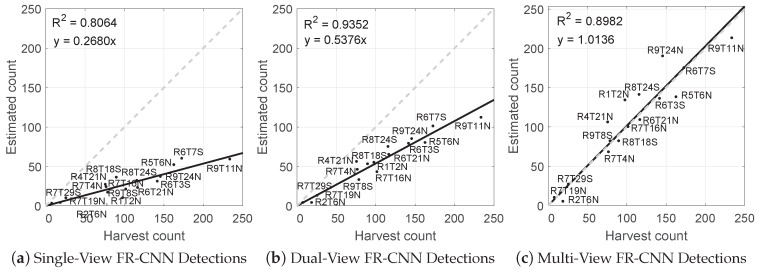
Linear models for the estimated number of fruits per tree compared to the harvest fruit count for: (**a**) single views of each tree (from the east); (**b**) dual opposing views; and (**c**) the proposed multi-view method. The line of equality is marked in grey.

Linear regression models with zero intercept (of the form y=mx) were fitted between the single, dual and multi-view estimates and the harvest fruit counts, as shown in Figure 10. The single-view method had the lowest precision ($R^{2}=0.81$) and lowest accuracy, counting only 27% of the fruit that was actually there. The dual-view method had the highest precision ($R^{2}=0.94$), but was also inaccurate, counting 54% of the fruit (double the single-view count). The proposed multi-view approach had high precision ($R^{2}=0.90$) and also high accuracy, counting 101.4% of the fruit (a 1.4% over-count). For the dual-view approach, the counting performance was additionally performed using human-labelled images to compare to the FR-CNN fruit detector. This required the full images of both sides of 16 trees to be labelled. It was not feasible to do the same test for the multi-view approach, because that would require more than 600 full images to be labelled. There was negligible difference in precision between the hand-labelled ($R^{2}=0.95$) and FR-CNN detections ($R^{2}=0.94$), but with human labelling, the percentage of detected fruit increased from 54% to 64%, which indicates that the FR-CNN detector missed some fruit in each image.

**Figure 11:**
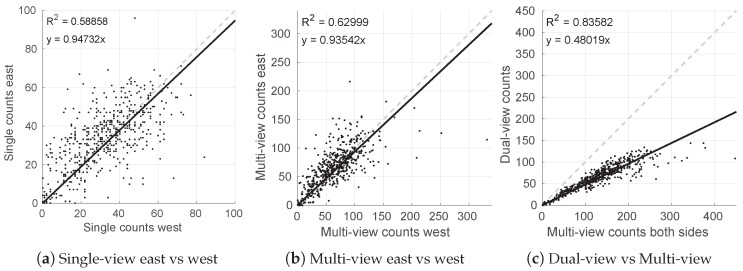
The relationship of fruit counts from opposing sides of every tree using (**a**) single-view and (**b**) multi-view approaches. The relationship between dual-view and multi-view estimates is shown in (**c**).

The single, dual and multi-view estimates were compared for all 522 trees, and the results are shown in Figure 11. Figure 11a,b show that there is significant variability in the number of fruits that are seen from the two opposing sides of the tree, whether using the single-view ($R^{2}=0.59$) or multi-view approach ($R^{2}=0.63$). This shows that individual trees did not distribute their fruit equally to both sides of the canopy, which also explains why the precision of the dual-view approach ($R^{2}=0.94$) was higher than the single-view method ($R^{2}=0.81$). The slope of approximately 0.94 in Figure 11a,b indicates a small bias; for this particular orchard block, slightly more fruit was observed on the west side.

A consistent relationship was observed between the dual and multi-view counts ($R^{2}=0.84$; see Figure 11c), and the slope was similar to that observed between dual-view and ground-truth data for 16 trees (0.48 compared to 0.54; see Figure 10b). Approximately 20 significant outliers (out of 522) can be seen in Figure 11c, where the multi-view approach appears to have over-estimated the count. It is more likely that the multi-view approach over-estimated than that the dual-view under-estimated, because the multi-view estimates are also outlying with respect to the distribution in Figure 14b. Similarly, four over-estimates were observed in Figure 9 and Figure 10c, when comparing the multi-view counts to the ground-truth.

### 3.4. Repeatability

**Figure 12:**
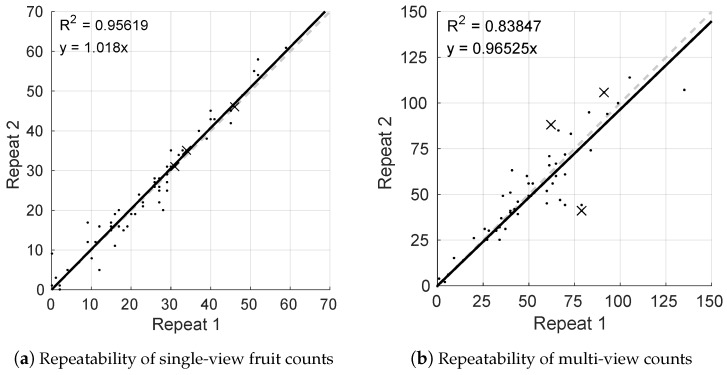
Repeatability of (**a**) single and (**b**) multi-view fruit count estimates for one side of orchard Row 5. A line is fitted to the data, and the respective $R^{2}$-value and slope are shown in the figure. The dashed line denotes the expected line of equality. Three significant outliers marked by crosses in (**b**) are investigated further in Section 4. They are included in the $R^{2}$ calculation.

One of the orchards rows (Row 5 west) was scanned twice on one side to evaluate the repeatability of the single and multi-view fruit estimates. As only one side was scanned twice, the dual-view repeatability was not evaluated, and the multi-view method was performed on the one side only. As the sum of two single-view estimates, the two-sided repeatability is likely to be slightly higher for both the dual-view and the proposed two-sided multi-view methods. The entire pipeline was re-run with the two datasets, including LiDAR segmentation and masking, FR-CNN fruit detection, fruit tracking and counting, and the estimates were compared for the two runs. The results are shown in Figure 12. Note that the magnitudes of the counts differ because in repeating just one pass from one side of the row, the multi-view approach tallies approximately half of the fruit on each tree, whereas the single-view approach tallies only the number of unoccluded fruit from the most central vantage point (27%, from above). The repeated single-view estimates had a slope of 1.02 and $R^{2}=0.96$, whereas the repeated multi-view estimates had a slope of 0.97 with $R^{2}=0.84$.

Both methods are accurate and repeatable (slope near unity), but the single-view method was more precise than the multi-view approach. The total row counts for the single-view approach were 1505 and 1532 mangoes, which corresponds to an error of 1.8%, whereas the totals for the multi-view approach were 3026 and 2981 mangoes, corresponding to an error of 1.5%.

### 3.5. Triangulation

**Figure 13:**
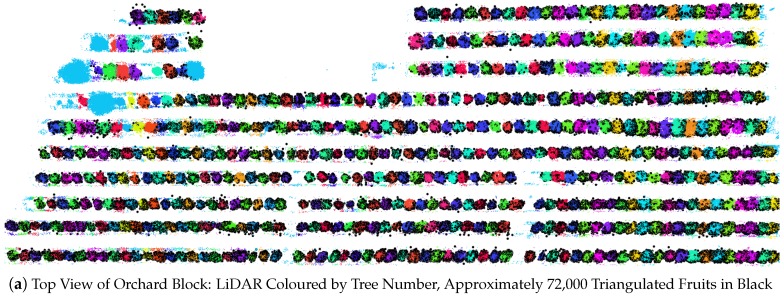

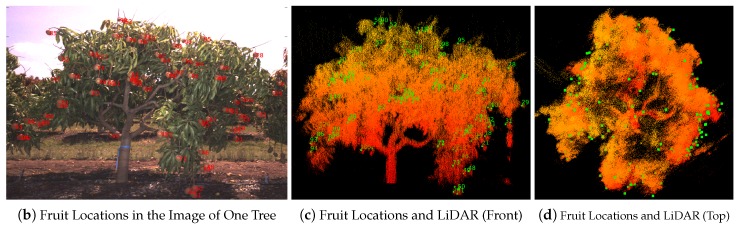
Triangulated 3D points of tracked fruit: (**a**) for the whole orchard block seen from above, with LiDAR coloured by tree number and triangulated fruit in black; (**b**) fruit locations and ids in a single image of one tree (r9t11n); (**c**) triangulated 3D fruit locations for the same tree, georeferenced with LiDAR data that are coloured by elevation and viewed from the same perspective as the image; (**d**) seen from above.

The correctness of the triangulated 3D fruit locations was assessed in two ways. Firstly, all fruits within a subset of four target trees were hand labelled for the entire multi-view sequences of images. Triangulation was performed using both the hand-labelled and FR-CNN detections and compared to assess the sensitivity of the positions to the detection method. For each fruit that was detected with FR-CNN, the nearest hand-labelled neighbour was found (in terms of the 3D Euclidean distance). For all fruit on the four trees, the mean nearest neighbour distance was 0.11 m, and the 95th percentile was 0.36 m. Secondly, the triangulated positions of all fruit in the orchard block (detected using FR-CNN) were georeferenced and viewed together with the LiDAR data, as seen in Figure 13. Qualitatively, good agreement was observed for all trees in the orchard block and at the scale of a typical individual tree.

### 3.6. Orchard Statistics

From the triangulated fruit locations, a number of spatial statistics were calculated, including:

- Spatial fruit-yield map
- Fruit totals per tree, row and block
- Histograms of fruit clustering (are the fruits evenly distributed within the canopy or not?)
- Histograms of fruit heights above the ground
- Histograms of canopy volume and relationship to yield

**Figure 14:**
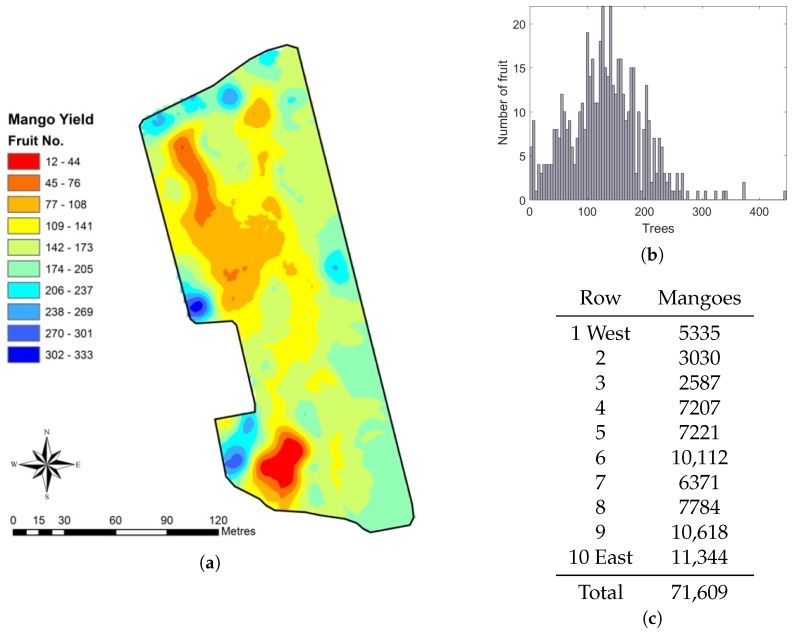
Spatial fruit statistics from the proposed multi-view method: (**a**) yield map of the orchard block, coloured by the number of fruit per tree; (**b**) histogram of fruit per tree; and (**c**) the estimated number of of mangoes for each row and the total. The first three rows have significantly lower yield because of the placement of a shed near the south end. The yield on the other seven similar length rows varies significantly, from 6371 to 11,344. The unproductive block in the southwest corner was removed after these experiments.

A yield map was created by averaging the triangulated geolocations of the fruit on each tree and mapping the total fruit count per tree at those locations. The total count estimates from the multi-view method were corrected by dividing by the slope of 1.0136 from Figure 10c. The result is shown in Figure 14, for a total of 71,609 mangoes. The map shows significant variability within the block. For example, Rows 6 and 7 are adjacent and similar in length, but the total counts varied from 10,112 to 6371 mangoes, respectively.

**Figure 15:**
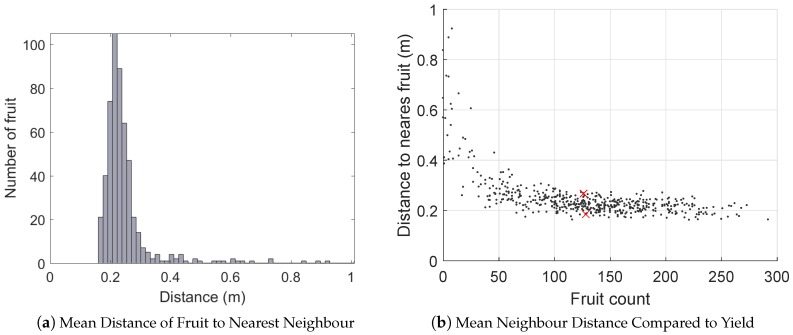

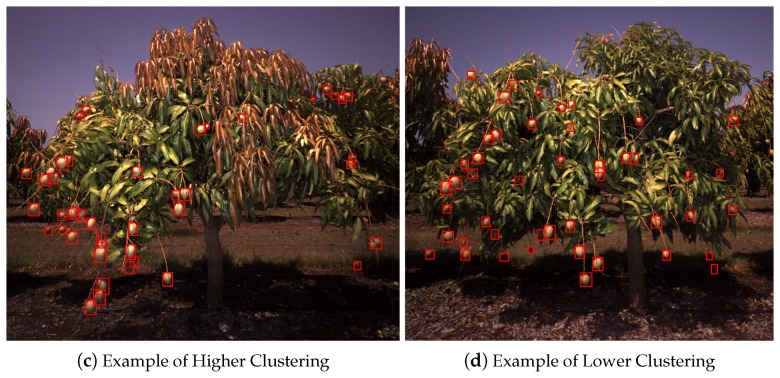
The clustering of fruit can be estimated by calculating the distance from each fruit to the next nearest: (**a**) histogram of nearest neighbour distances for all fruit in the orchard block. The relationship of mean neighbour distance to yield (**b**) follows a noisy inverse relationship. Two trees marked with a red cross have close to the average yield of 130 mangoes, with the minimum and maximum neighbour distances. These correspond to the the images in (**c**,**d**). In (c), the tree with the lowest mean neighbour distance shows a higher clustering, whereas (d) is the tree with the highest mean neighbour distance, showing a more even distribution of fruit in the canopy.

For each fruit, the distance to the next nearest was calculated to indicate the degree of fruit-clustering within the canopies. For example, a low mean nearest-neighbour distance indicates that the fruit are bunched within the canopy, which may impact the quality of the fruit. The results are shown in Figure 15. Figure 15a,b shows that there was significant variability in the neighbour distance, but that this is primarily a function of total yield (for similar sized canopy, more fruit implies tighter spacing). There is also a significant spread above and below the trend-line, due to the variable distribution of the fruit within the canopies. Figure 15c,d shows examples of minimal and maximal fruit clustering for two average yielding trees.

**Figure 16:**
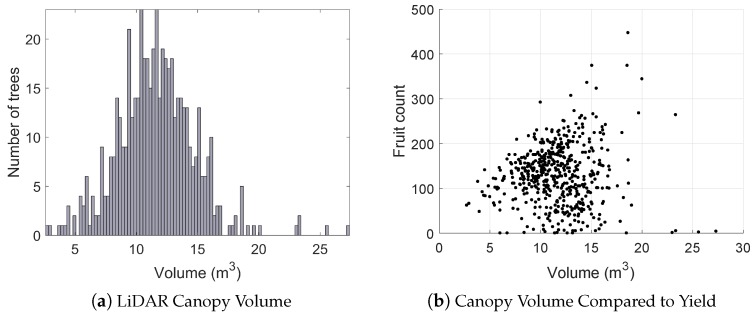
The LiDAR-voxel canopy volumes (**a**) and their relationship to yield (**b**) for all trees. Almost no relationship was observed between canopy volume and fruit yield.

**Figure 17:**
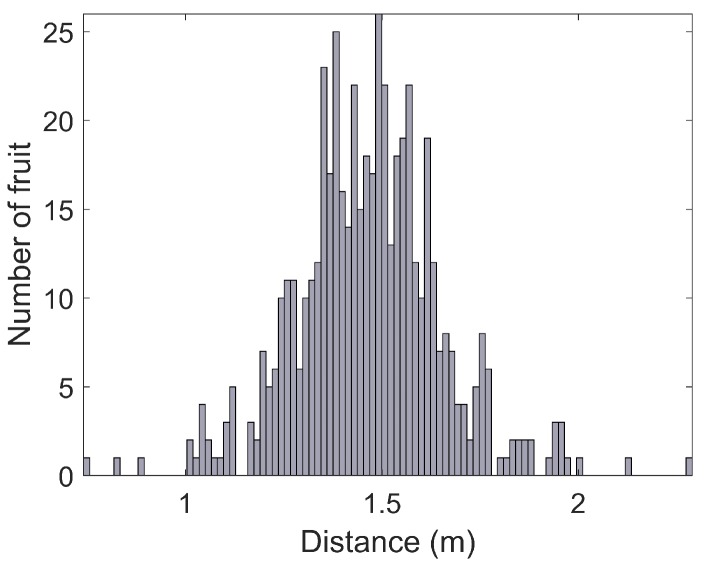
A histogram of the height of all fruit above the local ground surface, as measured by the LiDAR.

A histogram of canopy volumes calculated from LiDAR is shown in Figure 16, along with the relationship between canopy volume and yield (estimated by the vision system) for every tree. The results show that both the canopy volumes and fruit yields were variable, but that there was no discernible relationship between them. The height of each fruit above the ground is shown in Figure 17. The height distribution is reasonable considering the size and shape of the trees, but no ground-truth was available to quantify the accuracy of the height estimates for individual fruit. The histograms reported in this section were calculated for the entire orchard block, but this system readily permits evaluation at any scale. For example, fruit counts, clustering, heights above ground and canopy volumes can also be evaluated for each individual row or tree, which will be beneficial for defining decision support systems for improved per-tree agronomic management.

## 4. Discussion

In this section, the results are analysed to compare the performance of the the single, dual and multi-view approaches in terms of their utility for the practical application of counting fruit in an orchard. Additionally, we present further qualitative evidence to show how occlusion is addressed using multiple viewpoints and the identify some of the specific problematic scenarios that remain to be addressed as future work.

### 4.1. Performance of the Single, Dual and Multi-View Methods

Both the dual and multi-view approaches exhibited a very strong agreement with ground-truth fruit counts for the 16 target trees ($R^{2}\geq 0.90$), which indicates that both methods could be used as a means to map yield precisely per tree, per row and per block. The single-view method had reasonably strong agreement ($R^{2}=0.81$) and could also be used, but given the variability of the number of visible fruit seen from opposing sides of the tree ($R^{2}=0.59$), the dual-view method is preferable. Practically, the dual-view approach requires either two cameras (pointing to the left and right of the platform) or, as in our case, requires twice the traversal time and distance to scan both sides of the tree. In some cases, the single-view approach might have a cost benefit that justifies the reduction in performance.

Using hand-labelled images, the dual-view method observed 64% of the fruit, meaning that 36% of the fruit remain hidden due to occlusion (assuming that the human labellers did not miss any visible fruit). Using the FR-CNN detector, the dual-view method counted approximately half of the fruit, meaning that some visible fruit were missed by the detector in addition to the occluded ones that could never be detected with a single or dual-view approach. Nevertheless, the ratio of detected fruit to the actual total was very consistent ($R^{2}=0.94$) and could therefore be used as a yield estimator after calibration with the 16 target trees. Specifically, the dual-view output can be calibrated by dividing by the slope of 0.54 (from Figure 10b). The multi-view estimate had a similarly precise relationship to yield ($R^{2}=0.90$), but was much more accurate with a near unity slope of 1.014. The slope could similarly be used as a divisor after calibration; however, a 1.36% error is likely to be tolerable, to avoid the need for the labour-intensive calibration process.

Repeatability is a necessary, but not sufficient requirement for precision. The repeatability of the single-view method ($R^{2}=0.96$) was higher than for the multi-view method ($R^{2}=0.84$), although both methods are highly repeatable. When tallying one side of one whole row, the errors were similar and very low for both methods (<2%). That the single-view method was more repeatable than the multi-view method is of interest, because it suggests that if calibration is acceptable, then the dual-view approach (the sum of two single views) would lead to a more precise yield map than the multi-view method. The cause of several outliers is analysed in Section 4.4. The multi-view approach is algorithmically more complex and therefore has additional modes of failure. By contrast, the single or dual-view approaches are algorithmically simpler, but they strictly require the labour-intensive calibration step, which is also subject to human error. If any trees are manually miss-counted during calibration, it will affect all subsequent yield estimates. For the 16 target trees used in this study, the ratio of visible to total fruit was consistent. This is a requirement for calibrating the single or dual-view methods, but not for the uncalibrated multi-view method. The stability of this relationship is not known for other orchard blocks on the same farm, other orchards, different mango varieties, pruning regimes or for different fruits. We hypothesise that multi-view approaches will be more stable across these variables, but it remains as future work to prove.

### 4.2. Multiple Views as a Solution to Occlusion

The stable, near-unity gradient of the multi-view method supports the hypothesis that using multiple view-points is an effective solution to counting all of the fruit on the tree. A unity gradient could technically occur if the number of false positives and false negatives balanced perfectly, but this was not observed for the dual-view approach, which had a gradient of 0.54. The stable plateau of the gradient at one for widely-varying threshold values (see Figure 6) suggests that nearly all of the fruits on the tree were counted, including those hidden from the dual opposing views.

**Figure 18:**
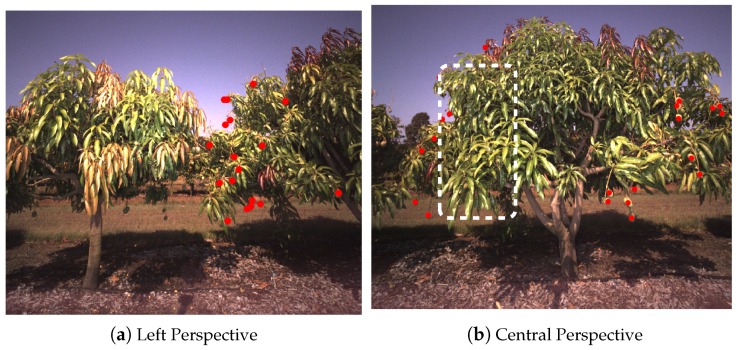
Fruit that was detected from the left perspective (**a**) of a tree is hidden from view in the central perspective (**b**), due to the occlusion of the foliage in the foreground. Red dots denote detected fruit, and the white rectangle marks the region of the tree where the hidden fruit would be.

To further support the argument, we manually examined dozens of sequences of images, detections and tracks to confirm both the correct behaviour of the tracker and the exposition of occluded fruit. A typical example is given in Figure 18, which shows the left and central perspective of a tree. Fruit that was detected in the left perspective was completely hidden from the central view. The multi-view detection and tracking approach allowed these fruit to be tallied, which led to the near unity gradient on average.

### 4.3. The Benefits of LiDAR

The proposed sensing system is multi-modal, relying on a GPS/INS, colour camera with strobes and LiDAR. The core system for fruit detection, tracking, counting and yield mapping requires only navigation and image data, whereas the LiDAR can be viewed as an optional extra. The use of LiDAR in this system provided the following advantages: association of fruit to specific trees, fruit height above the local ground surface and canopy volume. The triangulation of the fruit required only the navigation and image data, meaning yield mapping and tallying per-row or orchard-block can be done without LiDAR, but individual tree counts are only possible in the proposed system with the inclusion of LiDAR.

We observed a poor relationship between LiDAR canopy volume and yield. The volume information was therefore of no additional value for estimating yield in the orchard block used for our experiments. This further motivates the use of vision-based approaches that directly and explicitly detect and count fruit as a means to estimate yield, in preference to indirect observations and inference.

### 4.4. Problem Cases

This section highlights dominant modes of failure that adversely affected the tracking performance. These included missing image frames, platform oscillation, track sequence fragmentation, ambiguity due to fruit bunching and ground removal errors when generating the LiDAR masks. These were identified as primary sources of error during the design and development of the algorithm and will be addressed as improvements in future work.

#### 4.4.1. Missing Frames and Platform Oscillation

**Figure 19:**
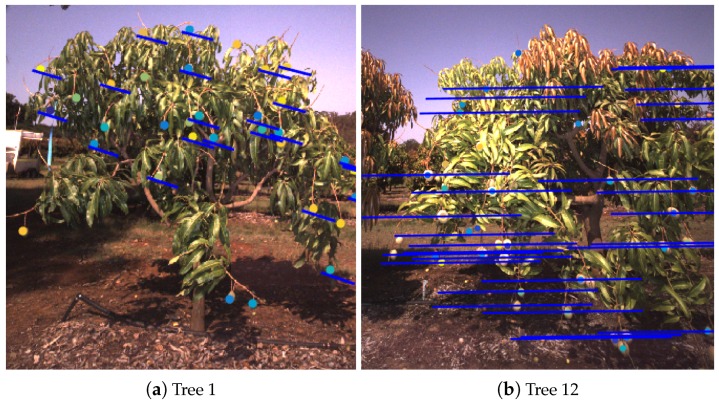
Examples of two trees where the base and repeated counts differed. The dots and the blue lines indicate tracked fruit and projections. When capturing the image in (**a**); the vehicle was oscillating rapidly. Immediately prior to capturing the image in (**b**), several images in the sequence were missing due to a logging error, resulting in longer projection lines.

The most significant errors occurred when epipolar lines were skewed or distorted, due to either missing image frames or platform oscillation. Three extreme multi-view repeatability outliers, which are marked by the crosses in Figure 12b, were inspected more closely to identify the cause of the discrepancy. The chosen trees were Tree 1, with repeated single-side counts of 62 and 88, Tree 12, with counts of 91 and 106, and Tree 60 with counts of 79 and 41. While driving past Tree 1, the vehicle hit a bump that caused the platform to oscillate rapidly. Despite optimising the time offsets between the navigation system and camera, the high rotation rates could not adequately be compensated for when generating epipolar lines. This is illustrated in Figure 19a. The direction of the epipolar lines compensated for the change in the vehicle pitch, but the rotation rate multiplied by any remaining time-synchronisation error caused a lateral pixel error which caused errors in tracking (a detailed mathematical analysis of the sensitivity of projection errors to time-synchronisation can be found in Underwood et al. [39]).

For Trees 12 and 60, during the repeat that produced the lower fruit count, the image sequences were missing in approximately half the images due to a logging error. This correctly resulted in much longer epipolar lines, as shown for Tree 12 in Figure 19b. The longer epipolar lines are geometrically correct, because the vehicle travelled further than normal between frames, but this resulted in too much ambiguity to accurately match and track all of the fruits in this case. Both error sources have already been corrected for our future experiments. The fault that caused missing frames was corrected by updating to the vendor’s latest application program interface (API). The navigation and camera system are now synchronised to sub-millisecond precision using the Network Time Protocol (NTP) with a pulse-per-second (PPS) between the GPS and computer and the Precision Time Protocol (PTP) between the computer and camera. This avoids the need to optimise the time-offset (as in Section 3.2), but that method is still required where hardware time synchronisation is not possible.

#### 4.4.2. Track Fragmentation and Over-Counting

**Figure 20:**
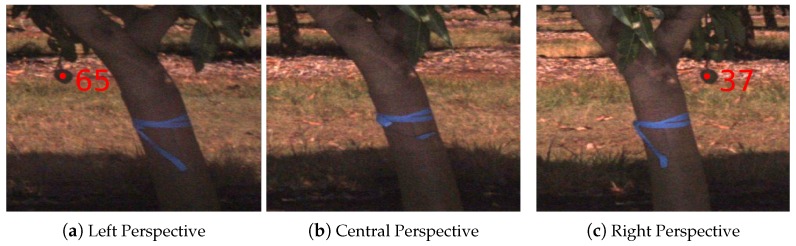
A single fruit is counted twice. The figure shows how a visible mango (65 in **a**) becomes occluded behind the trunk (**b**) and is counted as a new mango (37 in **c**) when reappearing.

Tracking is performed sequentially, from one image to the next. A track is only established when a mango is observed in at least two frames, which acts to filter false positives in the detection step. Conversely, a track is broken if the fruit is not matched, even if it becomes visible again later. This means that tracking is sensitive to a single false negative detection within the sequence, and it is sensitive to geometries that lead to alternating patterns of occlusion. A canonical example is shown in Figure 20. The mango was tracked until it was occluded by the tree trunk. It re-appeared, was tracked as a new piece of fruit and was therefore counted twice. The epipolar geometry permits matching from one frame to any other, but a policy would be required to avoid matching all permutations of image pairs (computationally intractable) and to avoid excessively long, ambiguous epipolar lines. One possible solution is to associate fruit in 3D after triangulation, which has been attempted for stereo data [4], but they found that GPS alignment errors from opposing sides were too large compared to the clustering of fruit.

#### 4.4.3. Fruit Bunching and Ambiguous Track Association

**Figure 21:**
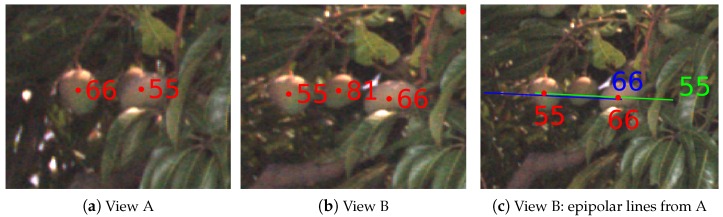
A false track association causes the identity of two mangoes to swap. In (**a**), two fruit are detected and assigned ids. In (**b**), the same two fruit are detected along with a third that is correctly assigned a new id. The corresponding projections are shown in (**c**). The ids of the two fruits have incorrectly swapped due to the similar distances to the epipolar projections.

The process of associating fruit between sequential pairs of images is purely geometrical, which means the mapping can become unclear when multiple fruits are bunched together. In Figure 21, an example is given where two nearby mangoes exchanged identifiers during tracking. The distances between the mango centroids and both epipolar lines were similar, as seen in Figure 21c, which led to the incorrect assignment in this case. In this scenario, the total count is not affected, but the incorrect assignment has an impact on the triangulation of the fruit. Triangulation uses the mean intersection of all rays corresponding to a fruit, so the magnitude of the error depends on the ratio of correct to false associations. Outlier rejection could potentially address the error during triangulation; however, a better approach would be to include triangulated depth information during track association. In the current system, a constant minimum and maximum range from the camera constrained the epipolar projections. Once a match occurs between two images, however, the actual range can be estimated separately for each fruit, and this could be used to refine the lengths of subsequent epipolar lines, to reduce the type of ambiguity seen in this case. Existing formulations could be adopted from conventional visual odometry, structure from motion and visual slam techniques to achieve this.

#### 4.4.4. Ground Extraction from LiDAR Masks and Counting Fallen Fruit

**Figure 22:**
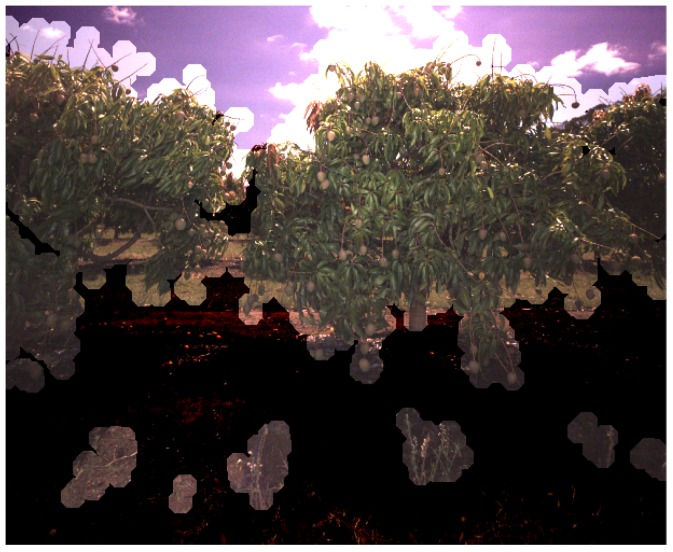
Tall grass (above 20 cm) is retained in the LiDAR data and projected when forming the image mask. Mangoes detected in these regions are likely to have fallen, but will still be counted.

When generating the LiDAR-based image masks, LiDAR points less than 20 cm above the local ground surface were deleted, so that the mask would not allow fallen fruit to be counted. In some cases, tall grass next to the tree, in the foreground of the image, was retained and projected into the camera frame, as seen in Figure 22. By chance, fallen fruit were sometimes detected in these regions and counted for that tree. Examples of fallen fruit can be seen in Figure 13b, as black dots outside of the canopies. Morphological operations could be used on the mask to remove the small disjoint regions seen in Figure 22. Future work will also explicitly tally fallen fruit around the base of each tree, as this may be of interest to growers. This is achievable with a minor extension to the proposed framework, whereby instead of deleting points below 20 cm, they can be retained as an additional layer in the mask. In each image, each detection will be assigned to one of the tree ids or to the ground layer beneath. This will provide an in-canopy and fallen fruit count for every tree.

## 5. Conclusions

In this paper, we introduced a multi-view approach to detecting, tracking, counting and locating fruit within a mango orchard. The primary objective was to address occlusion, which is a key problem in vision-based fruit counting. We conclusively demonstrated a practical solution to this problem, by combining images from multiple perspectives while driving past the trees. Further testing will be required to extend this conclusion to other varieties of fruit or pruning regimes. The method was rigorously tested on a commercially-relevant scale, and the results show that the system was able to count fruit with high precision and accuracy (1.36% error compared to ground-truth) even without the labour-intensive step of per-tree calibration. An extensive statistical evaluation was performed to compare single, dual and multi-view counting, which showed that all methods worked well with calibration, but only the multi-view approach could skip this step. The additional complexity of the multi-view approach resulted in an acceptably small reduction in repeatability compared to the simpler single and dual-view methods. The primary root causes of error were identified, along with the proposed remedies for future work. The advantages of the proposed system outweigh the additional complexity: the labour-intensive calibration process can be avoided, and every piece of fruit can be located in 3D, allowing a number of derived spatial statistics to be calculated.

## Figures and Tables

**Table 1:** Field and harvest mango count for 18 trees.

Tree	r9t11n	r9t24n	r9t8s	r8t24s	r8t18s	r7t4n	r7t16n	r7t29s	r7t19n
**Field count**	230	150	73	113	88	72	96	24	8
**Harvest count**	234	146	79	116	90	77	102	26	8
**Tree**	**r6t21n**	**r6t7s**	**r6t3s**	**r5t6n**	r5t14s *	**r4t21n**	r3t2n *	**r2t6n**	**r1t2n**
**Field count**	122	155	132	164	154	74	43	10	89
**Harvest count**	117	173	142	163	333	76	54	19	98

* Excluded trees: r5t14s had a discrepancy between manual field and harvest counts; r3t2n occluded by a neighbouring non-mango tree.
